# Sheptide A: an antimalarial cyclic pentapeptide from a fungal strain in the *Herpotrichiellaceae*

**DOI:** 10.1038/s41429-023-00655-6

**Published:** 2023-09-20

**Authors:** Robert A. Shepherd, Cody E. Earp, Kristof B. Cank, Huzefa A. Raja, Joanna Burdette, Steven P. Maher, Adriana A. Marin, Anthony A. Ruberto, Sarah Lee Mai, Blaise A. Darveaux, Dennis E. Kyle, Cedric J. Pearce, Nicholas H. Oberlies

**Affiliations:** 1 Department of Chemistry & Biochemistry, University of North Carolina at Greensboro, Greensboro, NC USA; 2 Department of Pharmaceutical Sciences, University of Illinois Chicago, Chicago, IL USA; 3 Center for Tropical & Emerging Global Diseases, University of Georgia, Athens, GA USA; 4 Mycosynthetix, Inc., Hillsborough, NC USA

**Keywords:** Structure elucidation, Parasite biology

## Abstract

As part of ongoing efforts to isolate biologically active fungal metabolites, a cyclic pentapeptide, sheptide A (**1**), was discovered from strain MSX53339 (*Herpotrichiellaceae*). The structure and sequence of **1** were determined primarily by analysis of 2D NMR and HRMS/MS data, while the absolute configuration was assigned using a modified version of Marfey’s method. In an in vitro assay for antimalarial potency, **1** displayed a pEC_50_ value of 5.75 ± 0.49 against malaria-causing *Plasmodium falciparum*. Compound **1** was also tested in a counter screen for general cytotoxicity against human hepatocellular carcinoma (HepG2), yielding a pCC_50_ value of 5.01 ± 0.45 and indicating a selectivity factor of ~6. This makes **1** the third known cyclic pentapeptide biosynthesized by fungi with antimalarial activity.

## Introduction

Malaria continues to be one of mankind’s most lethal diseases, causing an estimated 627,000 deaths and 241 million infections in 2020 alone [1, 2]. *Plasmodium falciparum*, one species of protozoan parasite causing malaria, is responsible for the most malaria infections in humans [3]. Unfortunately, *Plasmodium* species have shown resistance to core antimalarial drugs, including artemisinin and quinine, presenting a need for new antimalarial drug leads [4–6].

There are estimated to be millions of fungal species [7, 8], but fewer than 150,000 have been described taxonomically [7], and it is likely that even a smaller portion of these have been studied for the production of bioactive secondary metabolites [9]. With the goal of discovering antimalarial drug leads from filamentous fungi, a cyclic pentapeptide, sheptide A (**1**), was discovered from fungal strain MSX53339, a member of the family *Herpotrichiellaceae*, and shown to be active against *P. falciparum*.

**Fig. 1:**
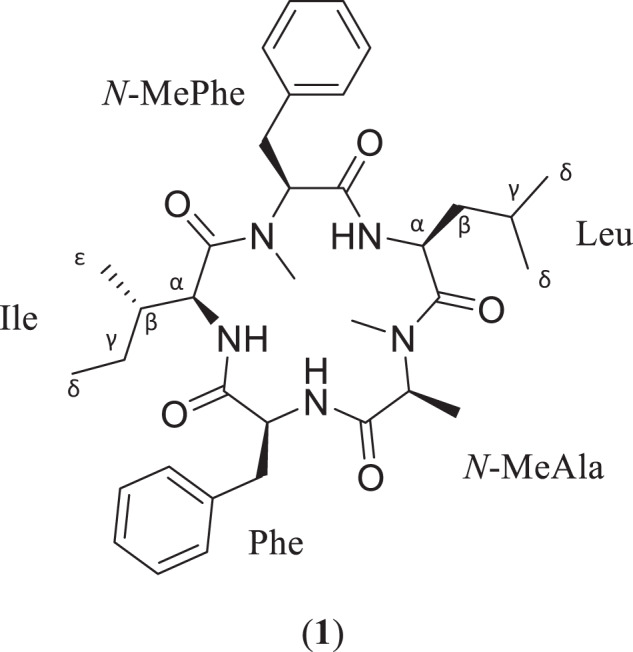
Structure and amino acid sequence of the cyclic pentapeptide, sheptide A (**1**)

At least fifty-seven cyclic pentapeptides have been isolated from a variety of fungal genera, such as *Aspergillus*, *Clonostachys*, *Fusarium*, *Hamigera*, *Penicillum*, *Pseudallescheria*, and *Xylaria* [10, 11]. This class of compounds has exhibited a wide range of biological activities, including antibacterial (avellanin A), antifungal [cyclo(l-Phe- l-Leu- l-Leu- l-Leu- l-Leu)], antiviral (aspergillipeptide D), chitinase inhibition (argadin), cytotoxic (cycloaspeptide F), immunosuppressive (pseudacyclin A), insecticidal (cycloaspeptide E), and vasoconstriction (avellanin A) activities [10, 12–14]. Currently, cycloaspeptides A and D are the only other cyclic pentapeptides isolated from fungi that exhibit antimalarial activity [10, 12–15], and we add to this via the discovery of **1** (Fig. 1).

## Results and discussion

An extract of fungal strain MSX53339 was studied as part of a new collaboration, where we are examining a diverse library of fungal cultures for antimalarial activity. A key goal is to identify leads that are more toxic to the *P. falciparum* parasite than they are generally cytotoxic to human cells [16, 17]. In this case, the lack of general cytotoxicity of this fungal extract (i.e. IC_50_ values > 20 µg ml^–1^ vs a panel of cancer cell lines) [18], coupled with promising dereplication data [19, 20], led us to further investigate the chemical diversity [21, 22] of this fungus for antimalarial potential.

Sheptide A (**1**) was isolated as a clear solid, and its molecular formula was determined as C_35_H_49_N_5_O_5_ by HRMS/MS, based on an *m/z* of 620.3787 (calcd for C_35_H_50_N_5_O_5_, *m/z* 620.3812) for [M + H]^+^, indicating an index of hydrogen deficiency of 14. The molecular formula matched that of the cyclic pentapeptides, persipeptides A and B, which were isolated from a *Streptomyces* species [23]. While there were similarities in the ^1^H and ^13^C NMR spectra of **1** and those of the persipeptides, **1** differed by three amino acid residues. Specifically, valine and *N*-methylvaline residues in the persipeptides were replaced by leucine, isoleucine, and *N*-methylalanine in the structure of **1**.

The ^1^H and ^13^C spectra of **1** (DMSO-*d*_6_; Table 1, Fig. S1) indicated the presence of seven methyls (including two *N*-methyls), three *N*-H protons, four methylenes, seven methines, two phenyls, and five carbonyls. Spectroscopy signatures for phenylalanine (Phe), *N*-methylalanine (*N*-MeAla), isoleucine (Ile), *N*-methylphenylalanine (*N*-MePhe), and leucine (Leu) were evident in the HSQC and H^1^-H^1^ TOCSY data (Figs. S2, S4). These findings accounted for 13 of the 14 degrees of unsaturation, with the final one resolved by the macrocycle.

**Table 1:** NMR data for **1** [700 MHz (^1^H) and 175 MHz (^13^C) in DMSO-*d*_*6*_]

Amino Acid	Position	^13^C	^1^H (mult., *J* in Hz)	HMBC	NOESY
Phe	C = O	169.9	–	–	–
	NH	–	7.14 (buried)	55.7, 168.8	2.32, 2.70, 2.80, 2.97, 3.28, 4.07, 4.17, 4.72
	α-CH	55.7	4.72 (m)	38.2, 169.9	7.91
	β-CH_2_	38.2	2.76 (dt, 14.3, 10.6) 2.97 (dd, 14.3, 6.9)	55.7, 129.4, 136.9, 169.9 55.7, 129.4, 136.9, 169.9	– –
	γ-C (ar.)	136.9	–	–	–
	*δ*-CH(1) (ar.)	129.4*	7.14*	38.2, 127.2*	2.32, 2.70, 2.80, 2.97, 3.28, 4.07, 4.17, 4.72
	*δ*-CH(2) (ar.)	129.0*	7.14*	38.2, 127.2*	2.32, 2.70, 2.80, 2.97, 3.28, 4.07, 4.17, 4.72
	2x ε-CH (ar.)	128.8	7.25	128.8*, 129.0*, 129.4*, 136.9	0.13, 3.36
	ζ-CH (ar.)	127.2	7.18	–	2.32
*N*-Me Ala	C = O	168.8	–	–	–
	N-CH_3_	29.7	2.32 (s)	55.9, 169.8	0.52, 0.86*, 1.17, 2.70, 4.17, 4.76, 7.14
	α-CH	55.9	4.17 (1, 6.9)	15.2, 29.7, 168.8	2.32, 4.76, 7.14
	β-CH_3_	15.2	1.17 (d, 6.9)	55.9, 168.8	1.34, 2.32, 2.70
Ile	C = O	170.8	–		–
	NH	–	7.91 (d, 8.7)	52.8, 169.9	0.13, 1.34, 2.32, 2.80, 2.97, 4.07, 4.72, 7.14, 7.34
	α-CH	52.8	3.36 (dd, 8.7, 8.2)	24.1, 36.7, 170.8	0.13, 0.52, 0.78, 4.07, 7.14
	β-CH_2_	36.7	1.34 (m)	12.1, 24.1, 52.8	3.36, 7.91
	ε-CH_3_	15.4	0.13 (d, 6.7)	24.1, 36.7, 52.8	0.52, 2.70
	γ-CH_2_	24.1	0.49 (m) 0.78 (dd, 18.8, 6.7)	– –	0.78, 3.36
	*δ*-CH_3_	12.1	0.52 (m)	24.1, 36.7	0.86*, 3.36
*N-*Me Phe	C = O	168.4	-	–	–
	N-CH_3_	31.3	2.70 (s)	61.7, 170.8	0.13, 0.52, 0.86*, 1.17, 2.30, 3.36, 4.07, 7.14, 7.34
	α-CH	61.7	4.07 (m)	31.3, 34.7, 137.8, 168.4	2.70, 3.36, 7.14, 7.34, 7.91
	β-CH_2_	34.7	2.80 (dt, 14.3, 10.6) 3.28 (buried)	129.4 61.7, 129.4, 137.8	7.14, 7.91 7.14
	γ-C (ar.)	137.8	–	–	–
	*δ*-CH(1) (ar.)	129.4*	7.14*	34.7, 127.2*	2.32, 2.70, 2.80, 2.97, 3.28, 4.07, 4.17, 4.72
	*δ*-CH(2) (ar.)	130.2*	7.14*	34.7, 127.2*	2.32, 2.70, 2.80, 2.97, 3.28, 4.07, 4.17, 4.72
	2x ε-CH (ar.)	128.8	7.25	128.8*, 129.0*, 129.4*, 137.8	0.13, 3.36
	ζ-CH (ar.)	127.2	7.18	–	2.32
Leu	C = O	169.8	–	–	–
	NH	–	7.34 (d, 9.4)	47.6, 168.4	2.32, 2.70, 4.07, 4.17
	α-CH	47.6	4.76 (d, 9.4)	169.8	0.86*, 1.49, 2.32, 4.17
	β-CH_2_	42.4	1.23 (dt, 13.3, 6.6) 1.79 (dt, 13.3, 7.1)	22.9, 47.6, 169.8 –	0.84*, 0.86*, 1.49, 2.70 0.84*, 0.86*
	γ-CH	24.8	1.49 (dq, 13.3, 6.6)	22.9, 42.4, 47.6	4.76
	*δ*_1_-CH_3_	22.9	0.84 (d, 6.6)	22.9, 24.8, 42.4	0.52, 1.23, 1.79, 2.32, 2.70, 4.76
	*δ*_2_-CH_3_	23.2	0.86 (d, 6.6)	22.9, 24.8, 42.4	0.52, 1.23, 1.79, 2.32, 2.70, 4.76

*Values can be interchangeable

**Fig. 2:**
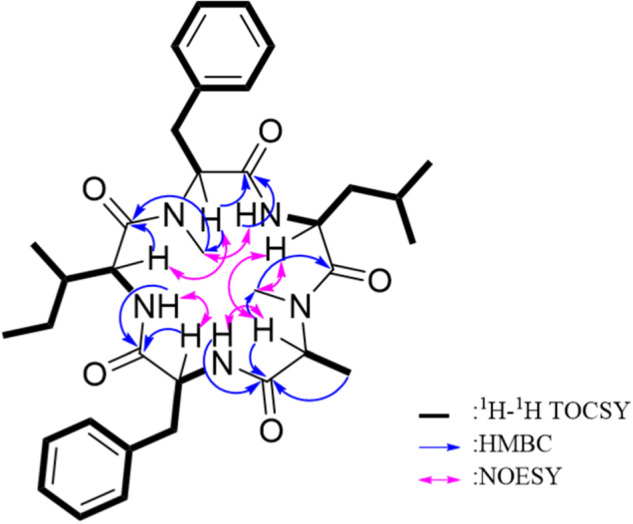
Key 2D NMR correlations for sheptide A (**1**)

The amino acid sequence was determined by extensive use of HMBC and NOESY correlations and further confirmed by HRMS/MS (Fig. S10). The presence of two phenyl and three aliphatic residues made deciphering the ^1^H spectrum of **1** (Fig. S1) more challenging due to peak overlap. A prime example was at 3.28 ppm, where one of the β-CH_2_ proton signals was obscured by a residual water peak at 3.30 ppm, such that the correlation was only evident in the HSQC spectrum. The second case was at 7.14 ppm, where a third *N*-H proton signal was masked by the phenyl signals, as determined using COSY and TOCSY correlations. Additionally, some ^1^H NMR signals that resembled complex multiplets were determined to be two overlapping signals, including the α-CH signals of Phe and Leu at 4.72 ppm and 4.76 ppm, respectively, the aliphatic β-CH_2_ signals of Phe and *N*-MePhe at 2.76 ppm and 2.80 ppm, respectively, and the *δ*-CH_3_ proton signals for Leu at 0.84 and 0.86 ppm. Based on HRMS/MS data, the initial sequence was determined to be *N*-MePhe^1^-Ile/Leu^2^-*N*-MeAla^3^-Phe^4^-Ile/Leu^5^ (Fig. S10). COSY and TOCSY correlations were used to confirm the spin systems of the individual amino acid residues (Fig. 2). Then, key HMBC and NOESY correlations were used to confirm their relative positions in the sequence.

Analysis of COSY and TOCSY spectra of **1** (Figs. S3, S4) was helpful in distinguishing the Ile and Leu residues, which can be challenging to differentiate, as they have the same monoisotopic mass and similar 1D NMR chemical shifts. The discerning feature between these residues is the presence of two nearly identical *δ*-methyl groups on Leu (*δ*_H_ 0.84 and 0.86 ppm), which resonate as two overlapping doublets in the ^1^H NMR spectrum (Fig. S1) that are both coupled to the γ-CH proton of Leu (*δ*_H_ 1.49 ppm) as determined by COSY and confirmed by a *J* value of 6.6 Hz. These methyl groups also show ^1^H-^1^H TOCSY correlations with the β−methylene protons and the α−proton (*δ*_H_ 1.23, 1.79, and 4.76 ppm respectively). In contrast, Ile has one terminal methyl and one secondary methyl, both of which show diagnostic chemical shifts and splitting patterns in the ^1^H NMR spectrum (Fig. S1). The terminal methyl (*δ*_H_ 0.52 ppm) had an indistinct splitting pattern, likely due to coupling with the two diastereotopic protons on the γ-methylene of Ile (*δ*_H_ 0.49 and 0.78 ppm). The secondary ε-methyl (*δ*_H_ 0.13 ppm) displayed as a doublet due to coupling with the adjacent β-methine proton (*δ*_H_ 1.34 ppm), which was also determined by COSY. Additionally, both methyls displayed ^1^H-^1^H TOCSY correlations with the other protons in the Ile spin system. In summary, distinguishing between Ile and Leu was possible after considering the data from multiple NMR experiments.

To confirm the amino acid sequence of **1**, key HMBC and NOESY correlations between respective residues were examined. Based on the HRMS/MS data, NMR confirmation of the peptide sequence started with the *N*-MePhe residue. An HMBC correlation between the NH proton of Leu (*δ*_H_ 7.34) and the carbonyl carbon of *N*-MePhe (*δ*_C_ 168.4) indicated that Leu was acylated by *N*-MePhe. This was supported by NOESY correlations between the NH proton of Leu (*δ*_H_ 7.34) and the *N*-CH_3_ protons of *N*-MePhe (*δ*_H_ 2.70). An HMBC correlation between the *N*-CH_3_ protons of *N*-MeAla (*δ*_H_ 2.32) and the carbonyl carbon of Leu (*δ*_C_ 168.4) was observed, indicating acylation of *N*-MeAla by Leu. This was confirmed by NOESY correlations between both the α-protons of *N*-MeAla and Leu (*δ*_H_ 4.17 and *δ*_H_ 4.76 respectively) and between the NH proton of Leu (*δ*_H_ 7.34) and the *N*-CH_3_ protons of *N*-MeAla (*δ*_H_ 2.32). This partial sequence fragment of *N*-MePhe^1^-Leu^2^-*N*-MePhe^3^ was confirmed by the HRMS/MS spectrum (i.e., *m/z* 360.2020). Next, an HMBC correlation between the *N*-H proton on Phe (*δ*_H_ 7.14) and the carbonyl carbon on *N*-MeAla (*δ*_C_ 168.8) indicated acylation of Phe by *N*-MeAla. NOESY correlations between the α-protons of Phe and *N*-MeAla (*δ*_H_ 4.72 and *δ*_H_ 4.17 respectively) and between the *N-*H proton of Phe and the *N*-CH_3_ protons of *N*-MeAla confirmed this connection. This was supported by a fragment in the HRMS/MS spectrum (i.e., *m/z* 507.2636), which was indicative of the aforementioned fragment plus the mass of Phe. This left the positioning of the Ile residue, which was placed between the Phe and *N*-MePhe residues, as confirmed by HMBC correlations between the *N*-H proton of Ile (*δ*_H_ 7.91) and the carbonyl carbon of Phe (*δ*_C_ 169.9), as well as, between the *N*-CH_3_ protons of *N*-MePhe (*δ*_H_ 2.70) and the carbonyl carbon of Ile (*δ*_C_ 170.8). This finding was buttressed by NOESY correlations between both the *Ν*−Η protons of Ile and Phe (*δ*_H_ 7.91 and *δ*_H_ 7.14 respectively) and between the α-protons of *N*-MePhe and Ile. Thus, the final cyclic peptide sequence was *N*-MePhe^1^-Leu^2^-*N*-MeAla^3^-Phe^4^-Ile^5^ (Fig. 1).

The absolute configuration of the amino acids was determined using a modified Marfey’s method [24]. After subjecting **1** to acid hydrolysis followed by derivatization with Marfey’s reagent, the 1-flouro-2,4-dinitrophenyl-5- l-alanine amide derivatives of each amino acid in **1** were subjected to LC-MS analysis along with derivatized standards of both d and l amino acids. Comparison of retention times and mass data revealed that the amino acid residues all had the l-configuration (Fig. S8).

Compound **1** was next evaluated as a potential drug lead for antimalarial activity, where it would be desirable to both kill the parasite but not be generally cytotoxic to eukaryotic cells. Against the parasite, *P. falciparum*, it displayed a pEC_50_ value of 5.75 ± 0.49. Alternatively, when tested in a standard counter screen for general toxicity against human hepatocellular carcinoma (HepG2) [25], moderate cytotoxicity was observed (pCC_50_ value of 5.01 ± 0.49). In examining the difference in anti-plasmodial vs cytotoxic activities, compound **1** exhibited approximately 6-fold selectivity for the parasite (Fig. S13). As a starting point for uncovering antimalarial leads, this level of selectivity is consistent with other fungal metabolites [26–29].

## Conclusions

A new cyclic pentapeptide, sheptide A (**1**), was isolated from a fungus of the family *Herpotrichiellaceae* (strain MSX53339) and was composed of the amino acid sequence *N*-MePhe^1^-Leu^2^-*N*-MeAla^3^-Phe^4^-Ile^5^. A suite of 2D NMR and HRMS/MS experiments were used to establish this sequence, and a modified Marfey’s method was used to determine the absolute configuration of the amino acid building blocks, which were all l. Compound **1** had approximately 6-fold selectivity for *P. falciparum* and represents the third reported cyclic pentapeptide of fungal origin with anti-plasmodial activity. Further studies are ongoing to modify the structure of **1**, with a goal of enhancing anti-plasmodial activity, minimizing cytotoxicity to eukaryotic cells, and developing a molecule that could be patented [30].

## Materials and methods

### General experimental procedures

Optical rotation, UV, and IR data were obtained using a Rudolph Research Autopol III polarimeter (Rudolph Research Analytical), an Agilent Cary Series UV-vis Spectrophotometer (Agilent Technologies), and a PerkinElmer Spectrum 65 FT-IR Spectrometer with Universal ATR attachment (PerkinElmer). NMR data were obtained using either a JEOL ECA-500 MHz NMR spectrometer operating at 500 MHz for ^1^H and 125 MHz for ^13^C (JEOL Ltd.) or an Agilent 700 MHz NMR spectrometer (Agilent Technologies, Inc.) equipped with a cryoprobe, operating at 700 MHz for ^1^H and 175 MHz for ^13^C. Residual solvent signals of DMSO (*δ*_H_ = 2.50 and *δ*_C_ = 39.5) were used as reference peaks. HRMS/MS data were collected via either a Thermo Fisher Scientific LTQ Orbitrap XL mass spectrometer or a Thermo Fisher Scientific Q Exactive Plus mass spectrometer, both equipped with a heated electrospray ionization (HESI) source (Thermo Fisher Scientific) and connected to a Waters Acquity UPLC system. A Phenomenex Kinetix C_18_ column (1.3 µm; 50 × 2.1 mm), heated to 40 °C, was operated at a flow rate of 0.3 ml min^–1^ with a gradient system of 15:85 to 100:0 of CH_3_CN-H_2_O (0.1% formic acid) over 10 min. MS data were collected from *m/z* 150 to 2000 in the positive mode. A Varian Prostar HPLC system, equipped with ProStar 210 pumps and a Prostar 335 photodiode array detector (PDA), was used to conduct all analytical and preparative HPLC experiments, with data collected and analyzed using Galaxie Chromatography Workstation software (version 1.9.3.2, Varian Inc.). All chromatography were conducted on Gemini-NX C_18_ analytical (5 µm; 250 × 4.6 mm), semipreparative (5 µm; 250 × 10 mm), or preparative (5 µm; 50 × 21.2 mm) columns (all from Phenomenex). Flash chromatography was performed on a Teledyne ISCO CombiFlash Rf 200 using Silica Gold columns (from Teledyne Isco) and monitored by UV and evaporative light-scattering detectors.

### Fungal strain identification and fermentation

Fungal strain MSX53339 was isolated in 1991 from leaf litter that was collected in a baboon sanctuary. Examination of cultural morphology grown on malt extract agar did not reveal any sporulating structures, making identification of this strain based on morphology ambiguous. Molecular sequences from the internal transcribed spacer region, ITS rDNA (specifically ITS1, 5.8 S, and ITS2), were obtained using primers ITS1F and ITS4 [31, 32]. Detailed methods for PCR and Sanger sequencing protocols were outlined previously [33]. A BLAST search in the NCBI database using the ITS region from the type and reference material [34] indicated relation to members of the family *Herpotrichiellaceae* Munk, in the order *Chaetothyriales*, Ascomycota. However, strain MSX533309 showed only ≥80% sequence homology with genera like *Capronia*, *Minimelanolocus*, and *Veronaea*. To further determine the phylogenetic disposition of this strain, we taxon sampled 10 of the 16 genera currently in the *Herpotrichiellaceae* [35]. Approximately 36 ITS sequences of *Herpotrichiellaceae* were obtained from a recent phylogenetic study [36] along with two outgroup taxa in the *Cyphellophoraceae*. Maximum likelihood analysis was implemented using IQ-TREE in PhyloSuite [37]. ModelFinder [38] was used to select the best-fit model using Akaike Information Criterion (AIC), and SYM + I + G4 was the best fit. Ultrafast bootstrapping was run with 5000 replicates [39]. Nodes with UFBoot ≥90% are shown on the clades, but only nodes ≥95% were considered strongly supported. Thus, strain MSX53339 was identified as a *Herpotrichiellaceae* sp. in the *Chaetothyriales*, Ascomycota (Figure S12). This strain is putatively a new genus or species within the *Herpotrichiellaceae*, but this hypothesis awaits further work due to the lack of morphological characters [40]. The sequence data were deposited in GenBank (ITS: OP207954 and OP207955).

The culture was stored on a malt extract slant and was transferred periodically. A fresh culture was grown on a similar slant, and a piece was transferred to a medium containing 2% soy peptone, 2% dextrose, and 1% yeast extract (YESD media). Following incubation (7 d) at 22 °C with agitation, the culture was used to inoculate 50 ml of a rice medium, prepared using rice to which was added a vitamin solution and twice the volume of rice with H_2_O in a 250-ml Erlenmeyer flask.

### Extraction and isolation

The solid-state fermentation culture was chopped into small pieces using a spatula, followed by the addition of 60 ml of 1:1 MeOH-CHCl_3_ and was then shaken overnight (~16 h) at ~125 rpm at rt. The resulting slurry was filtered in vacuo to form a filtrate, and the solid residue was rinsed with a small volume of 1:1 MeOH-CHCl_3_. To the filtrate, 90 ml of CHCl_3_ and 150 ml of H_2_O were added; the solution was stirred for 20 min before being transferred to a separatory funnel. The organic layer was collected and evaporated to dryness under vacuum using a rotary evaporator. The resulting organic extract was then partitioned between 100 ml of 1:1 MeOH-CH_3_CN and 100 ml of hexanes. The MeOH-CH_3_CN layer was collected and evaporated to dryness under vacuum. The defatted organic extract (~406 mg) was reconstituted in CHCl_3_ and absorbed onto Celite 545. The extract was then fractionated using flash chromatography with a solvent gradient of hexane-CHCl_3_-MeOH at a 30 ml min^-1^ flow rate and 61.0 column volumes to yield four fractions. Fraction 3 (~185 mg) was further separated into 11 subfractions using preparative HPLC with a solvent gradient increasing linearly from 40:60 to 55:45 CH_3_CN-H_2_O (acidified with 0.1% formic acid) over 6 min, followed by an isocratic hold at 55:45 CH_3_CN-H_2_O (acidified with 0.1% formic acid) for five minutes, and finishing with another linear increase from 55:45 to 100:0 CH_3_CN-H_2_O (acidified with 0.1% formic acid) over nine minutes, all at a flow rate of 21.20 ml min^–1^. Subfraction 9 yielded compound **1** (34.05 mg), which eluted at 21.5 min.

#### Sheptide A (1)

Compound **1** was isolated as a clear solid (34.05 mg); ${[\alpha ]}_{{{{{{\rm{D}}}}}}}^{20}$ = -80 (*c* 0.001, MeOH) UV (MeOH) λ_max_ (log *ε*) 204 (4.56) nm; IR (diamond) ν_max_ 3299, 2957, 1630, 1530 cm^-1^; ^1^H NMR (DMSO-*d*_6_, 500 MHz) and ^13^C NMR (DMSO, 125 MHz), Table 1 and Fig. S1; HR-HESIMS *m/z* 620.3787 [M + H]^+^ (calcd for C_35_H_50_N_5_O_5_, *m/z* 620.3812).

### Modified Marfey’s analysis

Approximately 0.2 mg of each amino acid standard was weighed into separate glass 2-ml reaction vials. To each standard was added 50 ml of H_2_O, 20 ml of 1 M NaHCO_3_, and 100 ml 1% Marfey’s reagent (*N-*α-(2,4-dinitro-5-fluorophenyl)-l-alaninamide) in acetone. The reaction mixtures were agitated at 40 °C for 1 h. The reactions were halted by the addition of 10 ml of 2 *N* HCl. The product of the reactions was dried under a stream of air and dissolved in ~1.7 ml of MeOH. Each derivatized standard was injected individually (0.7 ml) onto the UPLC. Also, aliquots of all the derivatized standards were combined to give a mixed standard, which was injected just prior to the digested and derivatized peptide **1**. UPLC conditions were 10–70% MeOH in H_2_O over 10 min on a BEH column and eluent monitored at 340 nm.

To generate the digested and derivatized peptide, approximately 0.2–0.3 mg of compound **1** was weighed into a 2-ml reaction vial, to which was added 0.5 ml of 6 *N* HCl. The compound was hydrolyzed at 110 °C for 24 h, at which time it was evaporated under a stream of air. To the hydrolysis product, 25 ml H_2_O, 10 ml 1 M NaHCO_3_, and 50 ml of 1% Marfey’s reagent in acetone were added. The reaction mixture was agitated at 40 °C for 1 h. The reaction was halted by the addition of 5 ml of 2 *N* HCl. The mixtures were dried under a stream of air and brought up in ~200 µl of MeOH and injected onto the UPLC with the use of the same conditions as for the standards.

#### Cytotoxicity assay

Human melanoma cancer cells MDA-MB435 and human ovarian cancer cells OVCAR3 were purchased from the American Type Culture Collection. These cell lines were propagated at 37 °C in 5% CO_2_ in RPMI 1640 medium, supplemented with fetal bovine serum (10%), penicillin (100 units/ml), and streptomycin (100 μg ml^-1^). Cells in log phase growth were harvested by trypsinization followed by two washes to remove all traces of enzyme. A total of 5000 cells were seeded per well of a 96-well clear, flat-bottom plate (Microtest 96, Falcon) and incubated overnight (37 °C in 5% CO_2_). Samples dissolved in DMSO were then diluted and added to the appropriate wells. Taxol (paclitaxel) was used as a positive control.

#### Antimalarial assay

A small aliquot of **1** was diluted to 10 mM in dehydrated, sterile DMSO (Tocris) and a duplicate-well, 12-point, 3-fold semilog dilution series was prepared at 1000× final concentration in 384-well plates (Greiner Bio-one) in DMSO using a Biomek 4000 (Beckman Coulter). DMSO was plated as the negative control and dihydroartemisin was diluted from 1 μM as the positive control. Source plates were sealed using foil sealing tape (VWR) and kept in a desiccator until used to inoculate *Plasmodium falciparum* assay plates. Compounds were assayed using methods previously described [25]. Briefly, *P. falciparum* clone W2 [41, 42] was cultured in RPMI (Gibco) supplemented with 10% inactivated human plasma (Interstate Blood Bank) and 5% hematocrit (Interstate Blood Bank) as previously described [43]. Assays were started by adding 40 µl of parasites at 2% parasitemia and 0.75% hematocrit to each well of 384-well plates (Greiner Bio-one) and then treated with **1** from the source plate using a 40 nl pin tool, resulting in a 1× test concentration. After incubation for 72 h, plates were fixed with 0.1% glutaraldehyde (Electron Microscopy Resources) and stained with Hoechst 33342 (Thermo Fisher Scientific) overnight before high content imaging (HCI) with a 4× objective on an ImageXpress Micro Confocal (Molecular Devices). Parasite DNA was quantified using built-in analysis software and normalized to positive and negative controls using CDD Vault. Compound **1** was tested in 5 independent experiments, and potency values listed represent the mean pEC_50_ (calculated as the -log EC_50_ [M]) from all replicates. To further investigate cytotoxicity, the same source plate containing a dilution series of compound **1** at 1000× was used to treat HepG2 cells (ATCC HC-8065) seeded at 2000 cells/well in the same type of 384-well assay plate (Greneir) for 72 h. At the endpoint, plates were fixed with 4% paraformaldehyde (Thermo Fisher Scientific) and stained with Hoechst 33342, so that cell nuclei counts could be assessed by HCI, as above. Compound **1** was tested in 4 independent cytotoxicity experiments and potency values listed represent the mean pCC_50_ (calculated as the -log EC_50_ [M]) from all replicates.

### Supplementary information

Supporting Information

## Data Availability

The NMR data for **1** were deposited in the NP-MRD (https://np-mrd.org/) under accession number NP0331808.
