# Supplementary material for: Sheptide A: an antimalarial cyclic pentapeptide from a fungal strain in the Herpotrichiellaceae
Source: J Antibiot (Tokyo). 2023 Sep 20;76(11):642–9. doi: 10.1038/s41429-023-00655-6 (PMC10602849; doi:10.1038/s41429-023-00655-6)
Supplement: Supplementary file 1 — Supporting Information [file 41429_2023_655_MOESM1_ESM.pdf]

Sheptide A: An Antimalarial Cyclic Pentapeptide from a Fungal Strain in the *Herpotrichiellaceae*

Robert A. Shepherd[1], Cody E. Earp[1], Kristof B. Cank[1], Huzefa A. Raja[1], Joanna Burdette[2], Steven P. Maher[3], Adriana A. Marin[3], Anthony A. Ruberto[3], Sarah Lee Mai[3], Blaise A. Darveaux[4], Dennis E. Kyle[3], Cedric J. Pearce[4], Nicholas H. Oberlies[1]

[1] Department of Chemistry & Biochemistry, University of North Carolina at Greensboro, Greensboro, NC, USA

[2] Department of Pharmaceutical Sciences, University of Illinois Chicago, Chicago, IL, USA

[3] Center for Tropical & Emerging Global Diseases, University of Georgia, Athens, GA, USA

[4] Mycosynthetix, Inc., Hillsborough, NC, USA

Table of Contents

Fig. S1. ^{1}H NMR spectrum of compound (**1**) [500 MHz, DMSO-d_6] and ^{13}C NMR spectrum of compound (**1**) [175 MHz, DMSO-d_6].

Fig. S2. HSQC NMR spectrum of compound (**1**) [700 MHz for ^{1}H and 175 MHz for ^{13}C, DMSO-d_6].

Fig. S3. Gradient Absolute Value COSY NMR spectrum of compound (**1**) [500 MHz, DMSO-d_6].

Fig. S4. ^{1}H-^{1}H TOCSY NMR spectrum of compound (**1**) [500 MHz, DMSO-d_6].

Fig. S5. HMBC NMR spectrum of compound (**1**) [500 MHz, DMSO-d_6].

Fig. S6. NOESY NMR spectrum of compound (**1**) [500 MHz, DMSO-d_6].

Fig. S7. HSQC-TOCSY NMR spectrum of compound (**1**) [700 MHz, DMSO-d_6].

Fig. S8. UPLC PDA chromatograms used for Marfey's analysis of compound (**1**).

Fig. S9. HR-HESI-MS spectrum of compound (**1**).

Fig. S10. HR-HESI-MS/MS spectrum of compound (**1**) with labeled fragments.

Fig. S11. UPLC-MS base peak chromatogram (top) and PDA chromatogram (bottom) of compound (**1**).

Fig. S12. Molecular phylogenetic analysis of strain MSX53339.

Fig. S13. Dose-response plots for potency and cytotoxicity determination of sheptide A (**1**).

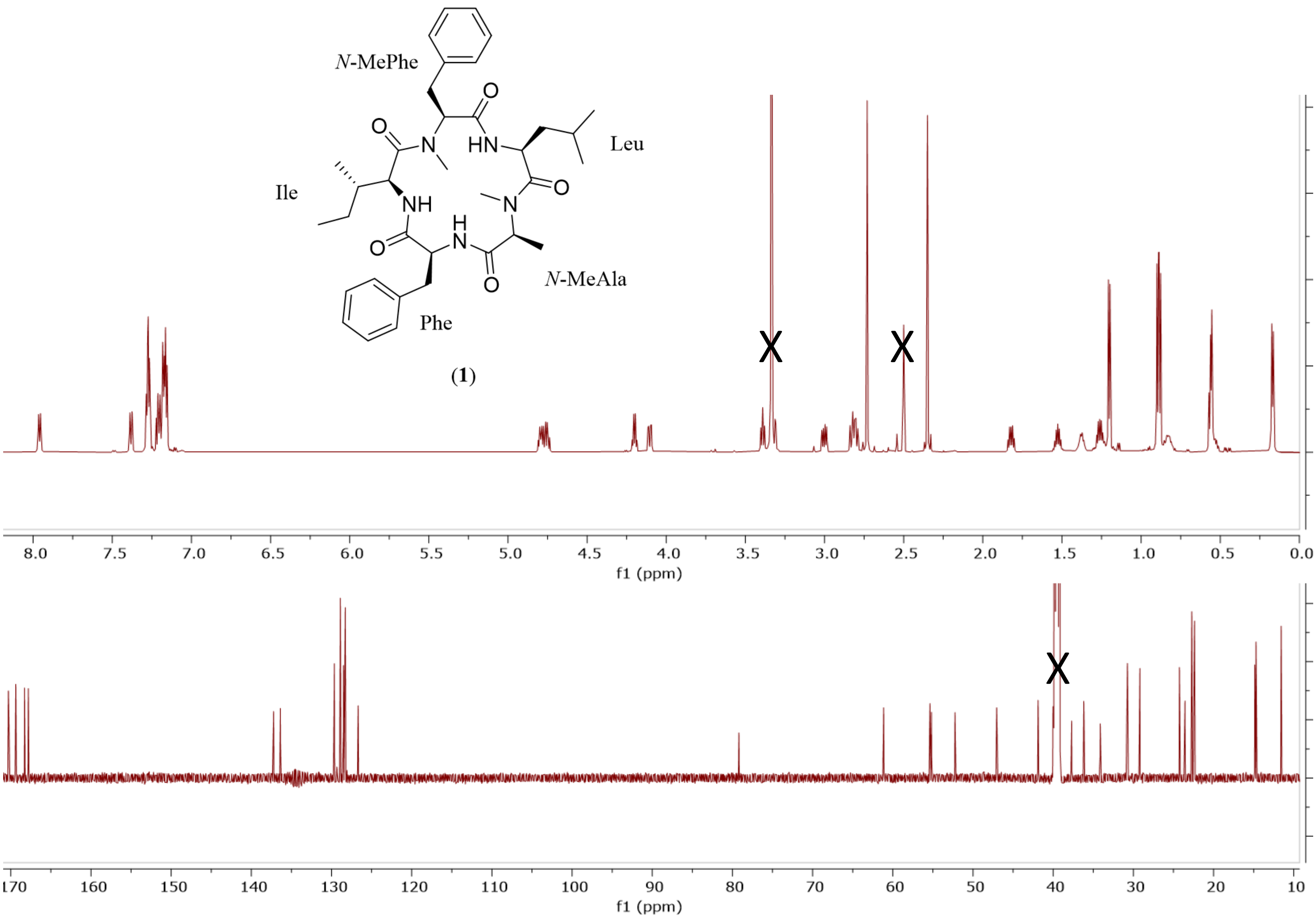

Fig. S1. ^{1}H NMR spectrum of compound (**1**) [700 MHz, DMSO-d_6] and ^{13}C NMR spectrum of compound (**1**) [175 MHz, DMSO-d_6].

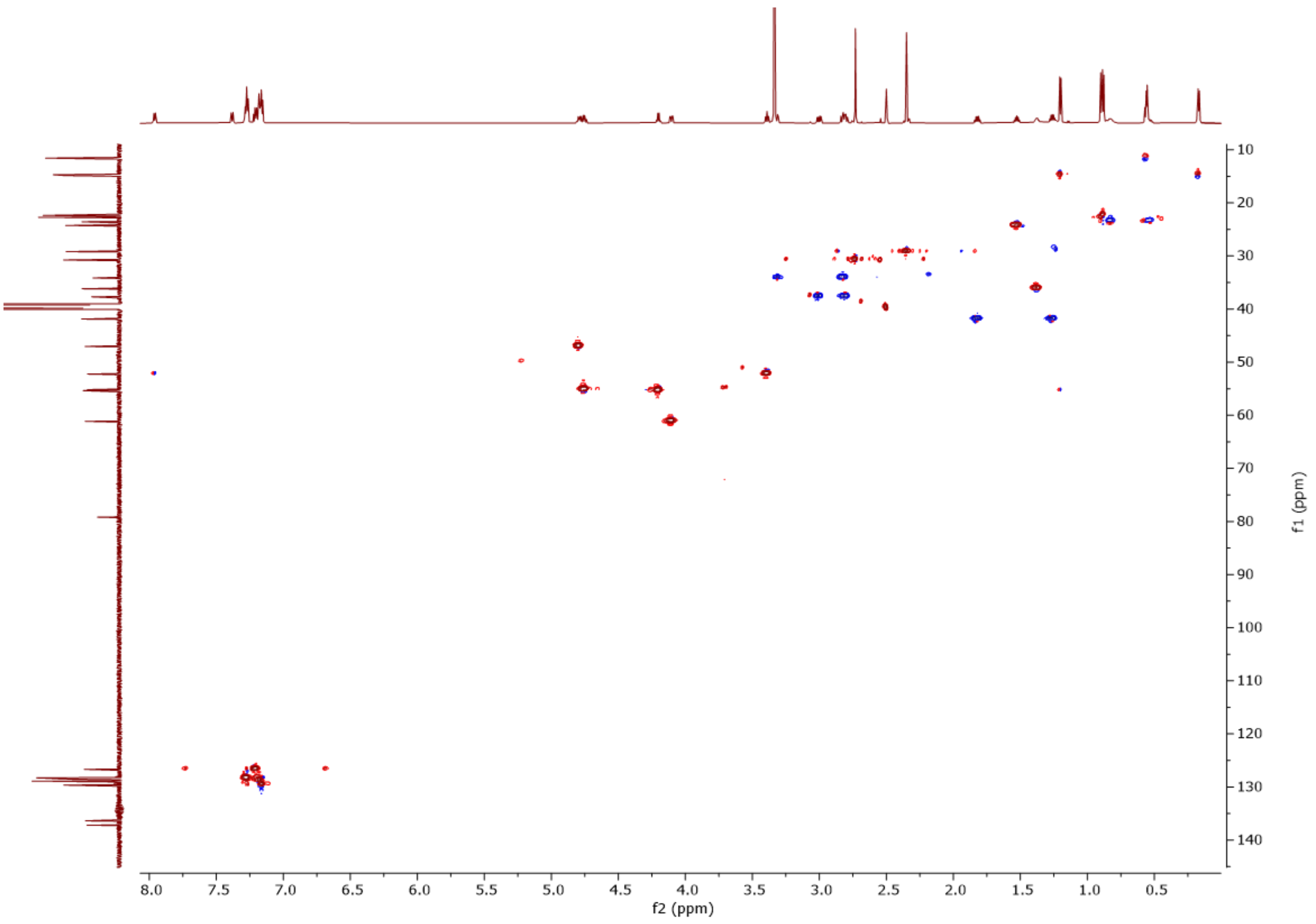

Fig. S2. HSQC NMR spectrum of compound (**1**) [700 MHz for ^{1}H and 175 MHz for ^{13}C, DMSO-d_6].

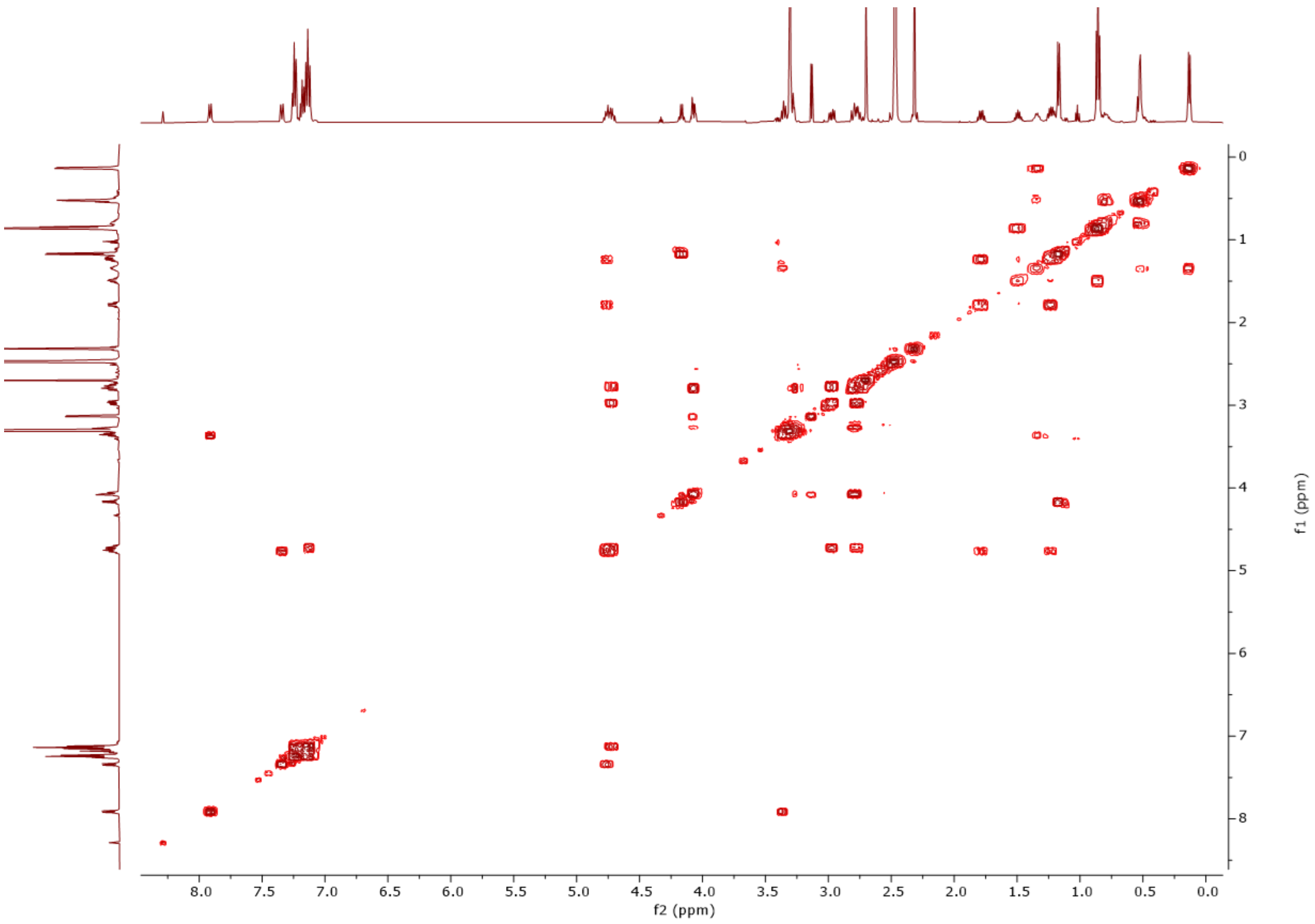

Fig. S3. Gradient Absolute Value COSY NMR spectrum of compound (**1**) [500 MHz, DMSO-d_6].

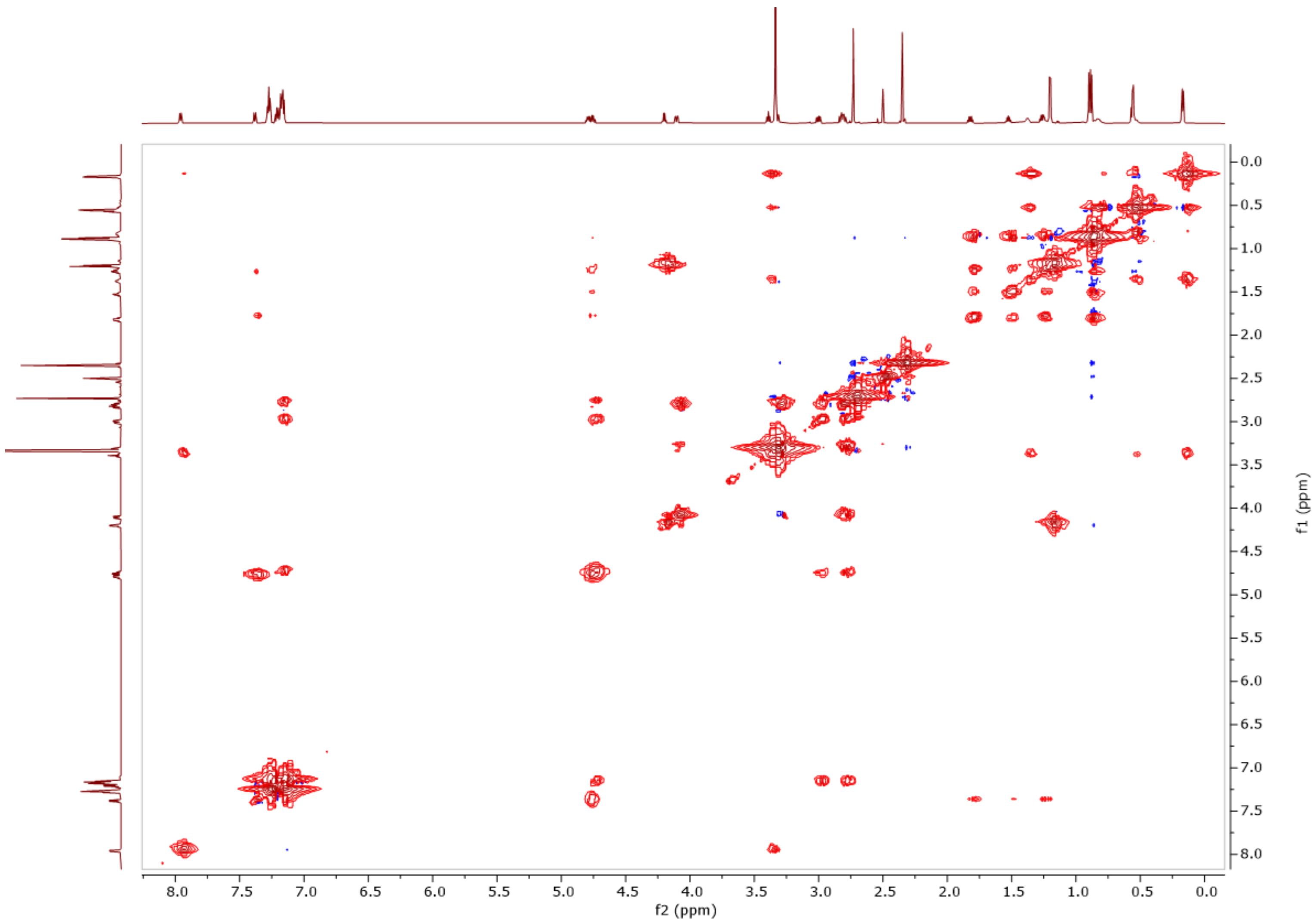

Fig. S4. ^1H-^1H TOCSY NMR spectrum of compound (**1**) [500 MHz, DMSO-d_6].

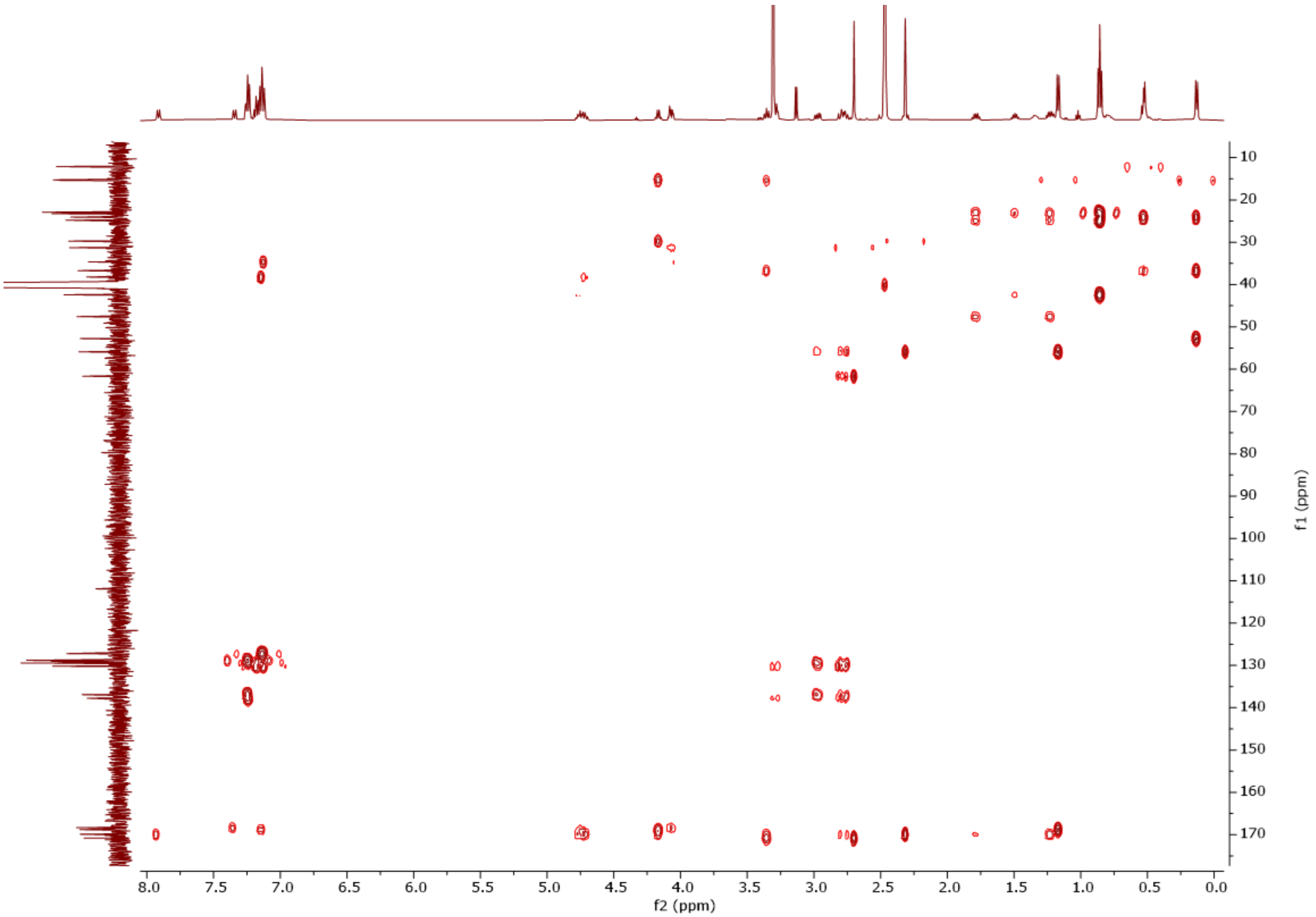

Fig. S5. HMBC NMR spectrum of compound (**1**) [500 MHz, DMSO-d_6].

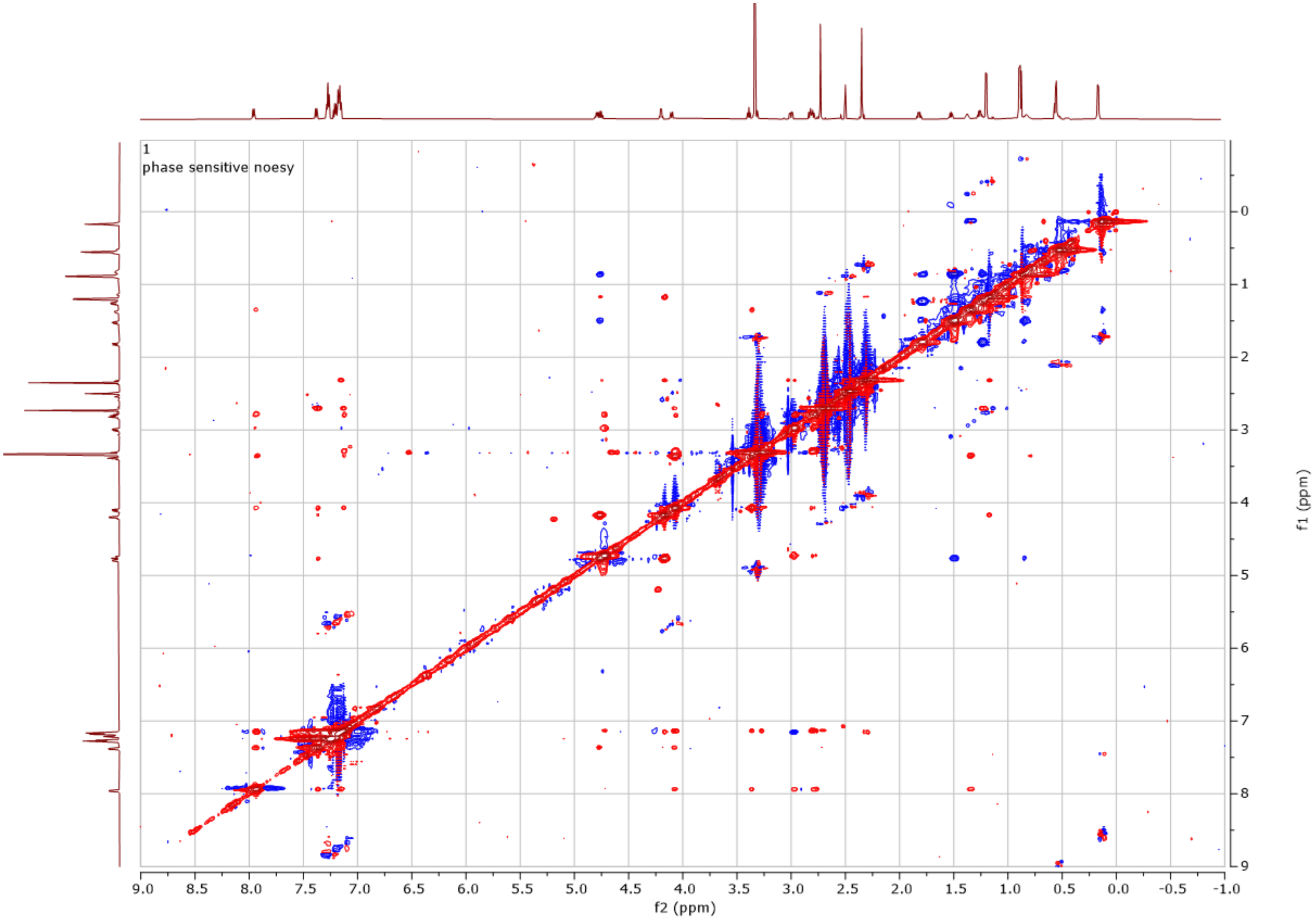

Fig. S6. NOESY NMR spectrum of compound (**1**) [500 MHz, DMSO-d_6].

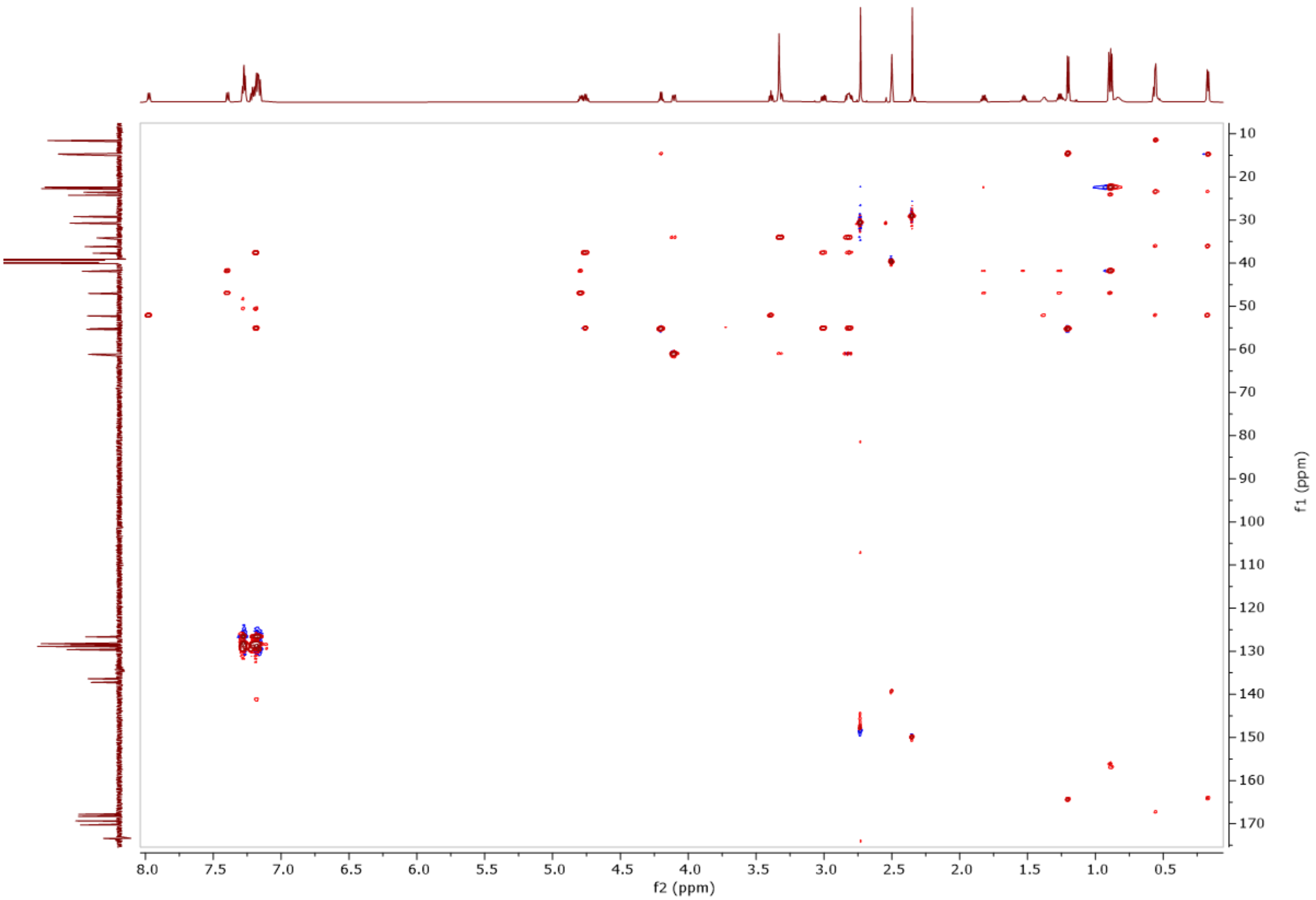

Fig. S7. HSQC-TOCSY NMR spectrum of compound (**1**) [700 MHz, DMSO-d_6].

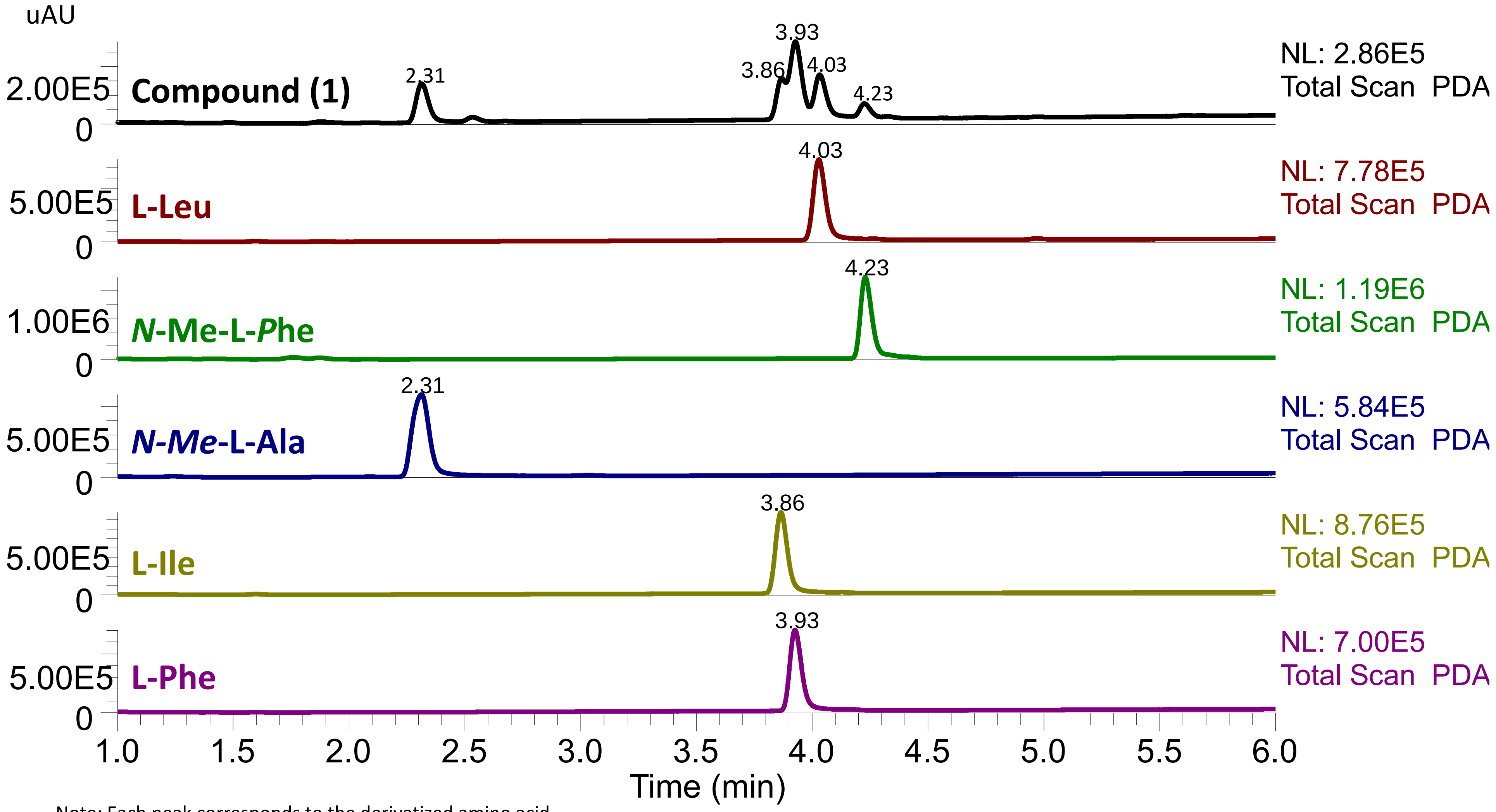

Note: Each peak corresponds to the derivatized amino acid.

Fig. S8. UPLC PDA chromatograms used for Marfey's analysis of compound (**1**).

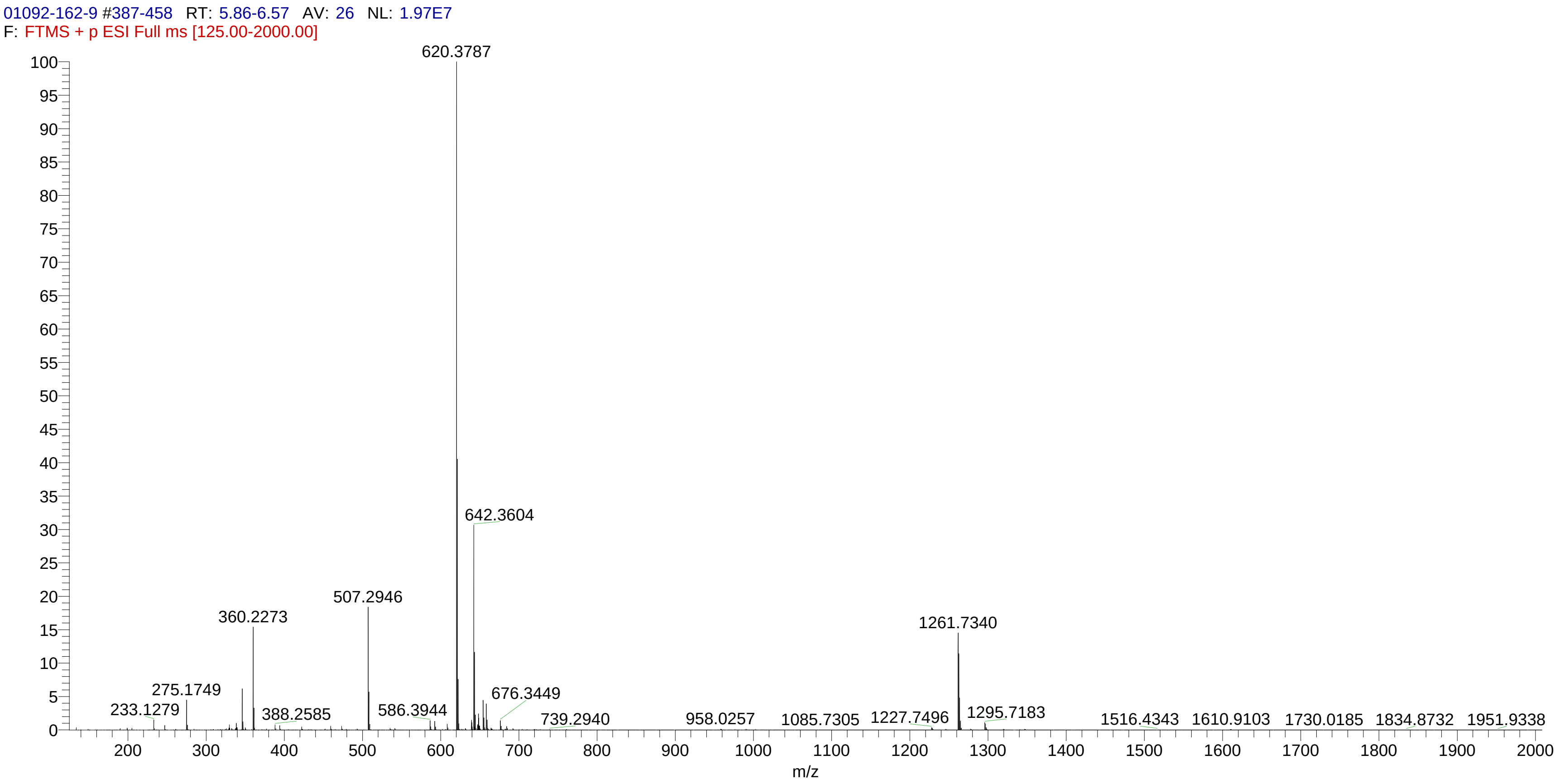

Fig. S9. HR-HESI-MS spectrum of compound (**1**).

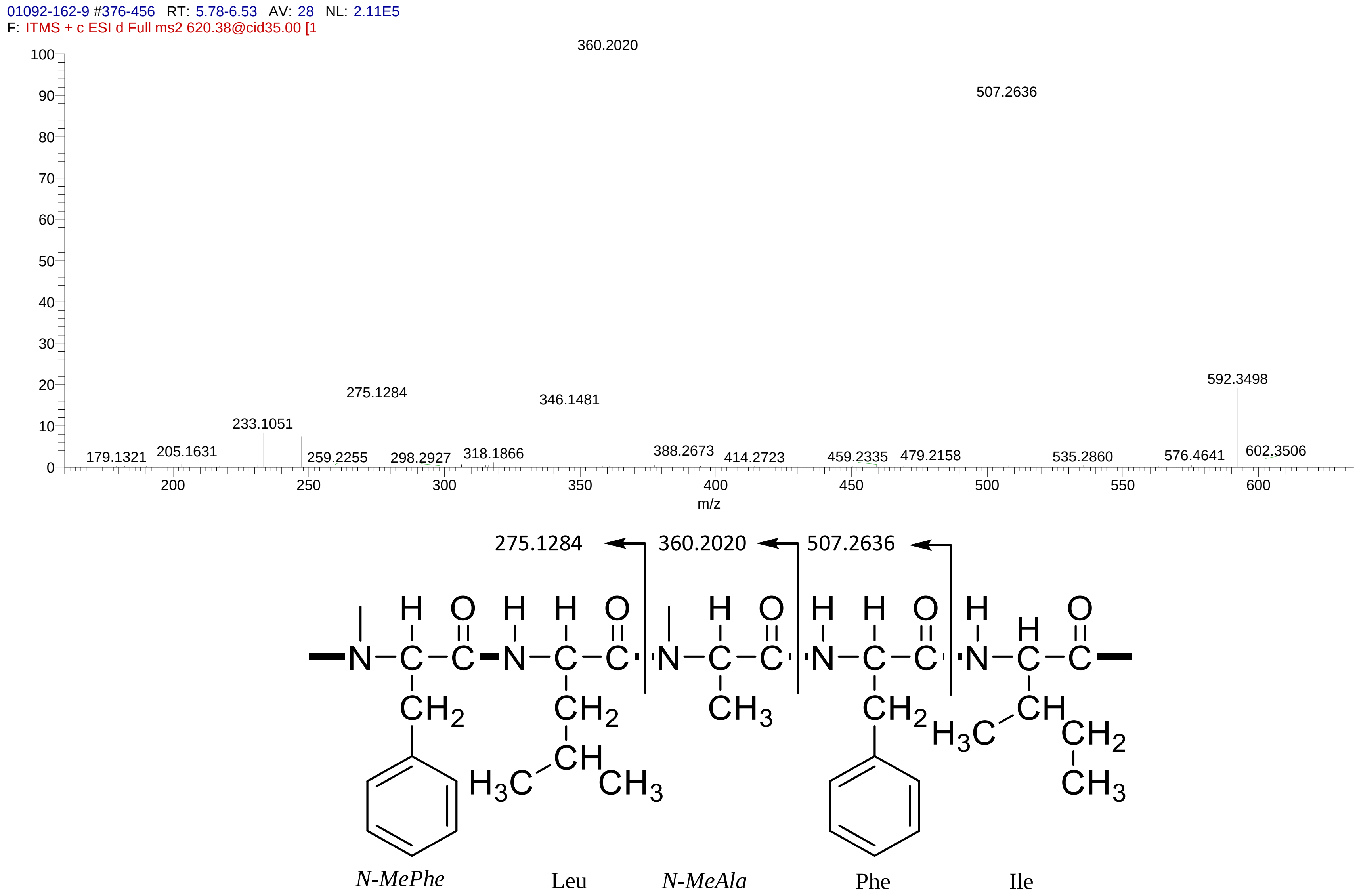

Fig. S10. HR-HESI-MS/MS spectrum of compound (**1**) with labeled fragments.

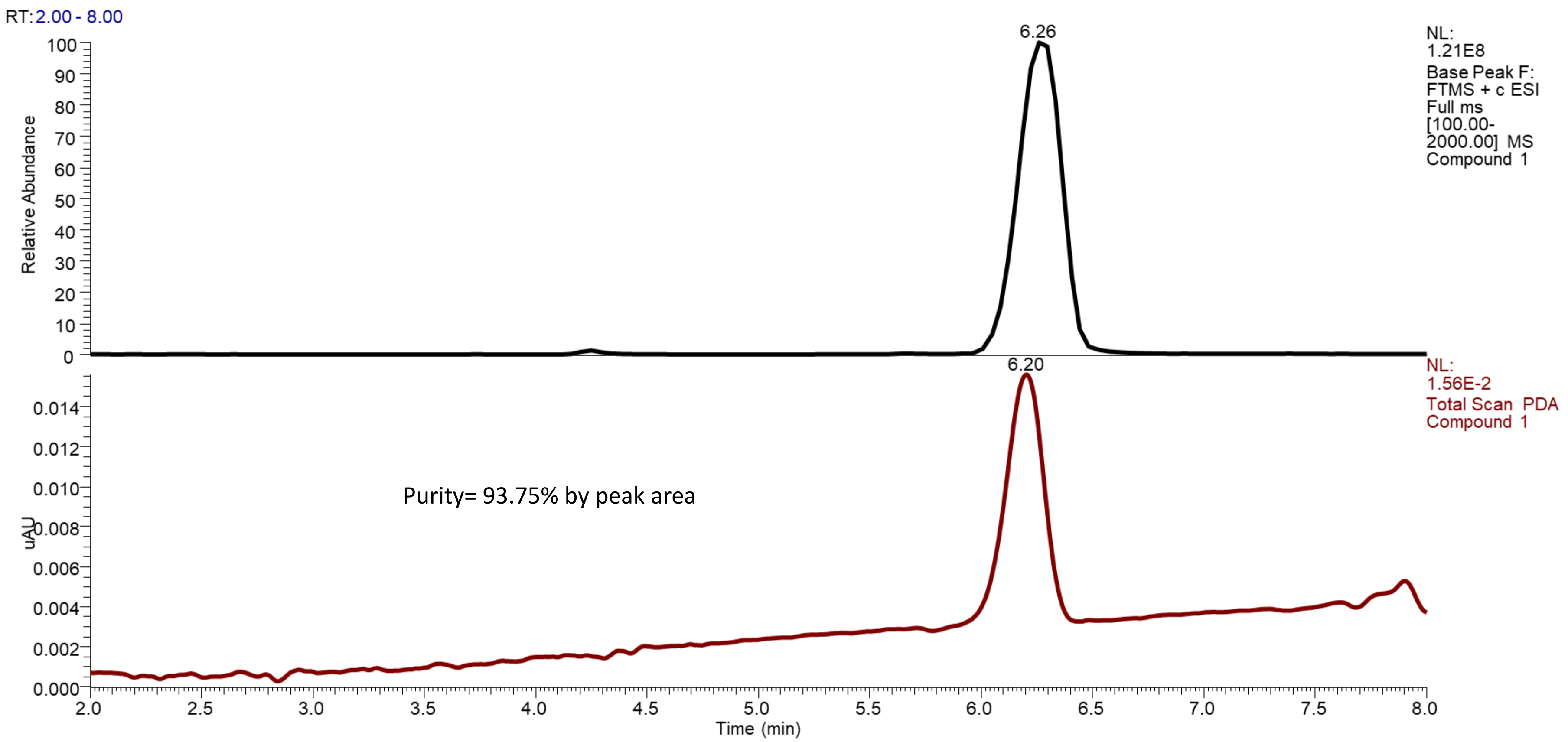

Fig. S11. UPLC-MS base peak chromatogram (top) and PDA chromatogram (bottom) of compound (**1**).

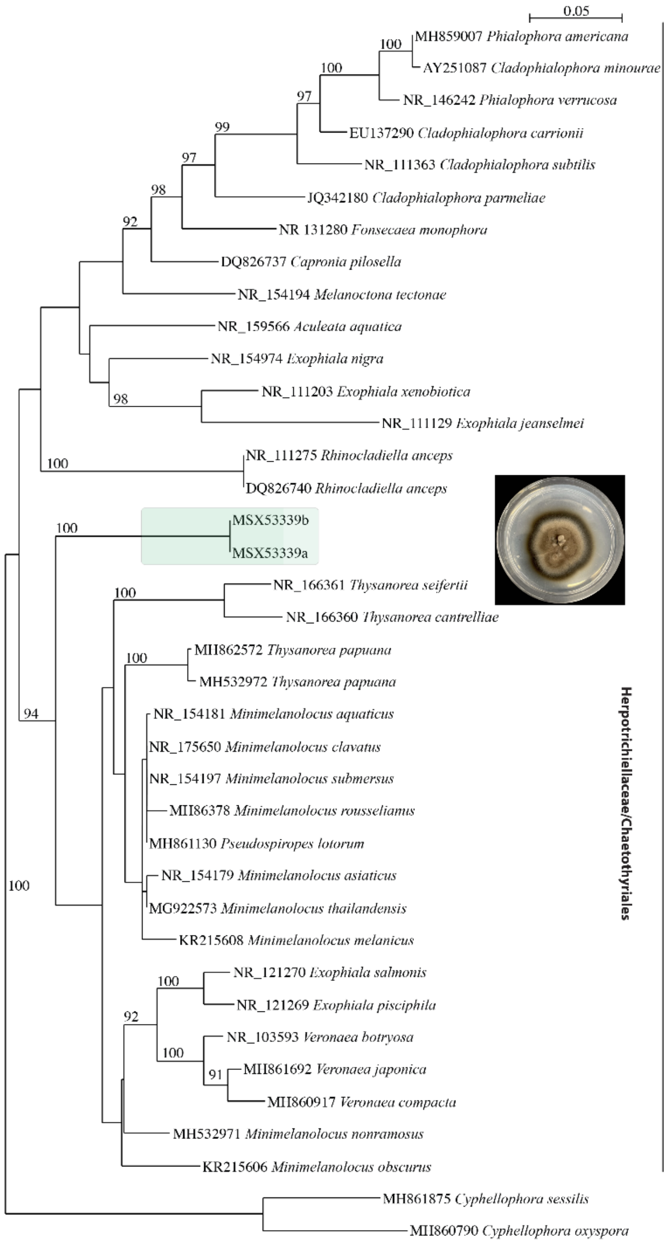

Figure S12. Molecular phylogenetic analysis of strain MSX53339 reveals phylogenetic affiliation to the family *Herpotrichiellaceae* Munk, *Chaetothyriales, Ascomycota*. Phylogram of the most likely tree (-lnL = 3500.819) based on ITS region using 38 sequences (453 bp). Maximum likelihood phylogenies were inferred using IQ-TREE under the SYM+I+G4 model for 5000 ultrafast bootstraps. Numbers refer to UFBoot support values ≥ 90 % based on 5000 replicates. Nodes ≥ 95 are considered strongly supported. *Cyphellophora sessilis* (MH861875) and *Cyphellophora oxyspora* (MH860790) were used as outgroup taxa. Bar indicates nucleotide substitutions per site. The prefix NR_ indicates sequences from type strains. Strain MSX53339 is highlighted in green, and a three week-old culture on malt extract agar is shown on the right.

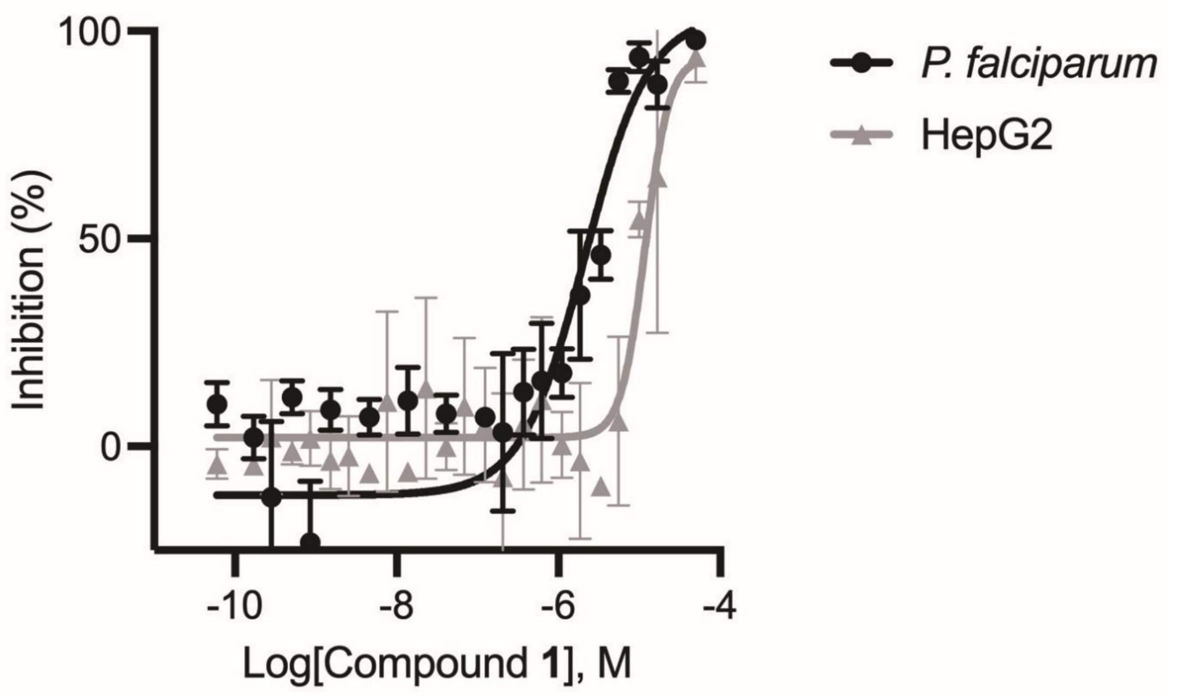

Figure S13: Dose-response plots for potency and cytotoxicity determination of sheptide A (**1**). Compound **1** was assayed against the *P. falciparum* W2 strain in five independent experiments, each with 2 replicate wells per dose, and against HepG2 cells in four independent experiments, each with 2 replicate wells per dose. Growth data were normalized to the positive (dihydroartemisinin) and negative (DMSO) controls for each run to calculate the percent inhibition for every well, then normalized data from all experiments were plotted together to calculate a single EC_{50} and CC_{50} to represent all replicates. Bars represent SEM.
